# Attachment, Mentalizing and Trauma: Then (1992) and Now (2022)

**DOI:** 10.3390/brainsci13030459

**Published:** 2023-03-08

**Authors:** Peter Fonagy, Chloe Campbell, Patrick Luyten

**Affiliations:** 1 Research Department of Clinical, Educational and Health Psychology, University College London, London WC1E 6BT, UK; 2 Faculty of Psychology and Educational Sciences, KU Leuven, B-3000 Leuven, Belgium

**Keywords:** attachment, trauma, developmental psychopathology, mentalizing

## Abstract

This article reviews the current status of research on the relationship between attachment and trauma in developmental psychopathology. Beginning with a review of the major issues and the state-of-the-art in relation to current thinking in the field of attachment about the impact of trauma and the inter-generational transmission of trauma, the review then considers recent neurobiological work on mentalizing and trauma and suggests areas of new development and implications for clinical practice.

## 1. Introduction

Thirty years ago, in a lecture delivered in 1992, the first author of this paper presented an overview of attachment and trauma and argued that attachment security and parental mentalizing may, at least to some extent, serve to “inoculate” children against the impact of exposure to traumatic life events, and that individual resilience might be understood in terms of those psychosocial processes. Since the publication of that lecture as a paper [1], the literature on both resilience and trauma has somewhat exploded, while the field of attachment research has developed crucial new insights and met fascinating challenges. The increasing focus on resilience and the promotion of resilience is a development that we are somewhat ambivalent about: an emphasis on salutogenic processes and the capacity to overcome difficulties should undoubtedly be supported, but where does that leave those individuals for whom such resilience is much harder to achieve? The bottom line is that we are still trying to answer the question of what is resilience/earned security of attachment and what makes trauma enduring.

In this paper, we will seek to develop a perspective on resilience that is framed less around individual strengths or weaknesses but instead sees resilience as the outcome of the extent to which an individual has experienced their social environment as one that supports trust, joint attention, and social learning. This paper thus seeks to reconsider the relationship between attachment, mentalizing and trauma, three decades on from the lecture mentioned above, in the context of major developments in neuroscience and mentalizing, and aims set out our current position in light of these developments.

We will begin by returning to attachment theory as the key theoretical foundation for the mentalizing model. We will provide a brief overview of what we regard as the most salient and pertinent recent research on attachment. We will then seek to establish how what we know now about attachment might inform an understanding of complex trauma and suggest that one of the damaging sequelae of such early experience is the disruption of epistemic trust (which we define as openness to the social communication of knowledge) and the capacity to meet with other minds in what has been termed the “we-mode”. We provide a summary of recent findings on the neuroscience of mentalizing, emphasizing that there is no one single mentalizing mechanism in the human brain: various sub-processes are involved in the many aspects of mentalizing that together constitute an experience of being and relating. Attachment style, we suggest, might be understood as a powerful factor in shaping this experience of self and other, sometimes influencing and sometimes being influenced by other expectations about the social environment and the extent to which that environment supports thinking and learning together. We partly seek to think about the position of attachment in this way because this field of work is at a somewhat vulnerable point: attachment is no longer a fashionable area of funding in developmental psychopathology. This is perhaps most powerfully encapsulated by the National Institute of Mental Health’s elimination of the Strange Situation from its Research Domain Criteria framework on the grounds that it is limited by reliance on a set of theoretical assumptions—namely, attachment theory—that are still widely debated [2]. Attachment thinking, understood most broadly, may partly have been a victim of its own success [3]. The significance of early relationships in supporting healthy development and protecting against maladjustment is now taken for granted, while the sophisticated hinterland of research and theoretical contributions that drove this shift is often overlooked. Meanwhile, concern has been raised, appropriately, about the ways in which attachment terms are used, for example by social workers in relation to child protection issues [4,5,6,7]. In the midst of these challenges, our objective here is to present our perspective on attachment, mentalizing and trauma and how they relate to difficulties in social function.

## 2. Intergenerational Transmission and the Internal Working Model: The Mechanism for the Generation and Loss of Resilience

One of the key premises of attachment theory is that attachment style can be transmitted from one generation to the next and that the sensitivity of the caregiver’s response to the infant shapes the attachment style that the infant develops. A caregiver who responds with sufficient contingency and accuracy—that is, with sufficient sensitivity—to the attachment signals displayed by the infant is more likely to foster a secure attachment style in the infant. A further key tenet of attachment research is that the attachment strategy adopted in the first few years of life, in response to the quality of care to which the infant has been exposed, influences that individual’s relational functioning into adult life: an “internal working model” is developed. This model for relating, based on the adult’s representations of their own attachment experiences in childhood, in turn shapes their own levels of sensitivity and responsiveness in parenting and thus the attachment experience of their children—creating the cycle of intergenerational transmission of attachment. Many studies of the intergenerational transmission of attachment have provided evidence supporting this process [8]. Particularly significant in relation to trauma is the idea that unresolved trauma in a parent may impact their capacity to respond sensitively to their infant to an extent that the parent (often non-consciously) reproduces the disorienting and/or frightening behavioral environment that they themselves experienced. As a result, the parent’s attachment trauma is revisited on and transmitted to the infant, described eloquently by Fraiberg et al. as “ghosts in the nursery” [9].

There are two major questions relating to the ideas of transmission and stability of attachment representations that have been the focus of attachment research over the past 30 years. The first concerns the “transmission gap”, which refers to consistent findings that caregiver sensitivity only partially mediates the robust relationship observed between caregiver attachment and child attachment [8]. The gap also includes a failure of sensitivity to account fully for the connection between unresolved parental loss and disorganized infant attachment [8].

The second issue is the relationship between infant attachment and later socio-emotional functioning and the continuity of attachment into adulthood—how significant is the internal working model in terms of its impact on social functioning across the life course? This continuity may enable us to understand attachment as a mediator in the well-established connection between early trauma and risk of psychopathology in later life [10,11]. Research on the association between attachment style and childhood disorders presents an emerging picture of the impact of attachment beyond infancy. Meta-analytic findings suggest that there is a significant positive relationship between disorganized attachment and externalizing behaviors in particular [12]. In another meta-analysis by the same group, all three categories of insecure attachment were roughly equal risk factors in relation to social competence in children [13]. In relation to internalizing symptoms, the association with attachment was considerably weaker, with avoidant children being found to be most at risk [14]. In recent years, there has been increasing interest in the role of child–father attachment in the developmental trajectory [15,16] and social and cognitive development [17,18,19,20,21] of the child. A meta-analysis found a moderate association between child–father attachment insecurity and externalizing behaviors and a smaller but still significant association between child–father attachment insecurity and internalizing behaviors [15].

Taking these meta-analytic findings together, the similar magnitude of associations suggests that attachment should be considered as coloring the network via which the quality of relationships to parents contribute to child development. A meta-analysis of nine studies using individual participant data to assess whether early attachment networks with mothers and fathers were associated with both internalizing and externalizing difficulties reported that children with an insecure attachment relationship with either or both parents were at higher risk of increased internalizing problems [22]. Secure attachment to either parent reduced internalizing behavioral problems only if the attachment to the other parent was secure. The findings also suggest that there is no significant difference between insecure attachment to the mother or the father in terms of increasing the likelihood of internalizing problems. It seems that the existence of an insecure attachment relationship within the child’s core network of interpersonal relationships is enough to result in heightened and more prolonged distress at times of need [23,24]. This leads insecurely attached children to have greater difficulty regulating their emotions and interacting appropriately with their peers, which in turn may further contribute to anxiety and other types of distress [25].

This individual participant meta-analysis [22] however found no association between secure and insecure attachment and externalizing behavioral problems, unlike previous meta-analyses [12], although there was a significant effect of insecure attachment to both parents on mother-reported externalizing behavioral problems. This was strongly driven by attachment disorganization only, rather than by either avoidant or anxious insecure attachment. Children with both disorganized and insecure attachment relationships with both of their parents had an increased risk of externalizing behavioral problems; in other words, the presence of one non-disorganized secure attachment relationship might protect them from behavioral problems. As in the case of internalizing behavioral problems, there was no difference in externalizing behavioral problems between children whose attachment to either parent was organized. The individual participant meta-analysis thus suggests stronger associations than previous meta-analytic findings between disorganized attachment and externalizing symptoms in terms of effect size [16]. The association between self-regulation and disorganized attachment [26], which is also consistently linked to externalizing problems in early life [27], may be part of an explanation for this association. A full understanding of the association may require consideration of the distinction between the disorganized-secure and disorganized-insecure types of categorization. As Dagan et al. [22] point out, this would require a large sample size, but it may be essential for fine-tuning potential etiological models, as the links between the two organized insecure attachment subcategories identified in meta-analytic and longitudinal studies have suggested different models for internalizing and externalizing behavioral problems [28,29].

While studies of attachment quality and outcomes emphasize the predictive value of the child’s network of attachments, the continuity of attachment classification across the life course suggests that there is significant (although again modest) stability [30]. For example, a large study of 850 participants comparing the Strange Situation conducted in infancy to the Adult Attachment Interview (AAI) carried out in late adolescence found significant but modest continuity [31]. A meta-analysis that updated previous systematic reviews designed to determine the stability of attachment classifications in early childhood (at infancy, in toddlerhood, and at pre-school/early school age), reported moderate stability at both the four-way (secure, avoidant, resistant, and disorganized; κ = 0.23) and secure/insecure (r = 0.28) levels of assessment [32], with secure attachment organization appearing to be the most stable. The stability of attachment classifications across the lifespan may partly reflect the constancy of the environment rather than the durability of early relationships, a point made 40 years ago [33]. 

Research with adoptive families, where the child’s environment changes markedly and mostly in a positive way, offers an important source of information about the impact of early attachment throughout the lifespan [34,35]. Findings over the past decade have suggested that children with experiences of sometimes quite severe maltreatment or institutionalization in early life can show considerable improvement in the quality of their attachments after being adopted. Most children placed in new family environments form attachments to their new caregivers within a year [36,37] and show significant recovery. There is a more strongly increased likelihood of attachment security in adopted children than in children whose environment is not changed [38].

However, children with a history of early adversity are still substantially less likely to form secure attachments to their adoptive parents than non-adopted children are to their parents [39], as assessed with story stem methods [40,41,42], autobiographical narratives [43], and family drawings [44]. These findings suggest that thoughts and feelings about close relationships are likely to be somewhat affected by early experience regardless of later attachment relationships. Adoptive children’s attachment security within the new parent–child relationship does not reflect maltreatment or institutionalization, as indicated by (perhaps more bias-prone) parent-reported attachment behaviors [45,46,47] and the reports of adoptees concerning their felt security in their relationships with their adoptive parents [48,49]. However, the experience of early adversity can have lasting repercussions for adult attachment representations. Thus, while there was no difference between a group of 22–25 year olds who had experienced severe deprivation in orphanages in Romania and a group of young adults who had been adopted but had not experienced early deprivation in terms of the quality of their relationships with their adoptive parents [50], general representations of attachment on the AAI had higher rates of insecure (non-autonomous) states of mind [51] in the deprived group. This finding suggests that maltreatment in childhood confers a risk for non-autonomous states of mind in adulthood, regardless of adoption status [52]. New environments generate fresh relationship representations independent of early experiences, suggesting the existence of discontinuity of attachment patterns and the non-deterministic nature of early experience. Yet, these fresh relationship-specific representations are probably created on top of more abstract, global attachment representations, which can continue to reflect early experience.

While the intergenerational transmission of attachment is well established in intact parent–child dyads [8], there may be good reasons to question whether this intergenerational concordance is mediated by genetic factors shared by parents and their children rather than the influence of attachment-related states of mind on parenting behavior [53,54]. Evidence from twin studies suggests that attachment patterns in adolescence may be shaped by genes rather than the shared parenting environment: the largest study of twin attachment found that at age 15, 40% of variance in security was hereditary, whereas studies of twins have consistently suggested a limited role of heredity in attachment in infancy [55]. Research with adoptive families allows the genetically informed study of the process of intergenerational transmission of attachment [34]. Early studies indicated a psychosocial model of transmission whereby, despite a lack of genetic relatedness, there was high agreement between the attachment states of mind of foster mothers and their infants’ patterns of attachment [56]. More recent studies yielded mixed results [57], and a meta-analysis of these studies found that the association between parents’ attachment states of mind and their young children’s attachment patterns depended on the biological relatedness of the parents and their children [8].

Both the intergenerational transmission of attachment and the continuity of attachment representation across the individual’s lifespan are being increasingly understood within the context of ecological risk factors. A meta-analysis of 4396 parent–child dyads found that effect sizes for the transmission of autonomous-secure representations to secure attachments were smaller under risk conditions and in adolescents, whereas the transmission effect was greater for older children [58]. Simulation studies similarly indicate that the stability of attachment seems to be largely dependent on the stability of the environment [59]. The position that attachment should adjust in response to contextual changes is, rather than a challenge to the field, in keeping with attachment theory’s origins in evolutionary thinking: attachment is best understood as an interpersonal strategy to optimize adaptation to a particular environment.

To summarize, we know that the intergenerational transmission of attachment is a real effect that is at least partially explained by parental sensitivity and, more specifically, we know that unresolved trauma transmits a higher risk of disorganized attachment. We also know that there is some continuity in terms of the stability of attachment and that the likelihood of stability is confounded by ecological risk. The more sophisticated picture of attachment provided by these findings is not as starkly predictive as the popular schema of attachment might envisage. However, given the immensely complex and multifactorial nature of human development, the consistency and significance of this body of findings is of great value. To return to the National Institute of Mental Health’s criticism of attachment-informed measures as being derived from assumptions that are widely debated, it could be argued that in comparison to many developmental models, attachment should be considered pre-eminent precisely because it does have a theory and because its key assumptions have been tested and replicated so many times. In support of the model of the transmission of attachment, for example, a meta-analysis has indicated a significant positive association between insecure adult attachment and maltreatment in childhood [60], and, further, unresolved attachment appears to mediate the link between maltreatment in childhood and maladjustment in adolescence [61].

## 3. Thinking about Trauma

In this section we will consider how the mentalizing model can enrich our understanding of the attachment perspective on trauma. There is, for example, evidence that high levels of caregivers’ reflective functioning with regard to their own traumatic experiences might be a protective factor in moderating the effects of parental early adversity on offspring outcomes [62]. Higher reflective functioning in relation to trauma in parents with a history of childhood sexual abuse and neglect was shown to be associated with a lower risk of attachment disorganization and a substantially lower risk of exposure to childhood sexual abuse in their own offspring [63]. Such works highlight the need for more direct tests of the role of parental reflective functioning in the association between parents’ attachment history and outcomes for their children [64].

We also seek to link the mentalizing approach to research on adversity and its implication in inequalities in mental health both within and between countries. The well-known findings of the Dunedin Multidisciplinary Study found that nearly 80% of economic burden among adults was associated with 22% of the population. The high-needs segment of the population could be identified with high accuracy from age 3 years, and four risk factors were identified for these children: growing up in a socioeconomically deprived family, being exposed to maltreatment, having a low IQ, and having poor self-control [65]. Adverse childhood experience is a social phenomenon. Its prevalence differs widely across societies [66] and, within socioeconomic groups, there are differing levels of deprivation [67]. To understand the developmental trajectory associated with the cumulative disadvantage identified in the most vulnerable population group, attachment theory might need to expand beyond the dyad to consider not only how early experiences might generate an internal working model for close relationships, but also how early social experiences generate a capacity for social learning and the social connectedness that comes with it.

Disorganized attachment has real effects: as mentioned above, research indicates that it is associated with poorer mental health outcomes [12]. Furthermore, disorganized attachment is as strongly associated with deprivation as it is with trauma [68]. A meta-analysis of over 6000 infants suggested that mothers’ higher socioeconomic status along with greater maternal sensitivity and higher metacognition during pregnancy predicts lower risk of attachment disorganization in their children [69]. These findings are only partially consistent with the expectation that the association between socioeconomic deprivation and disorganization is an outcome of the reduced capacity for sensitive parenting in parents who are facing sustained and multiple stressors.

It may be instructive to go back to some of Mary Main’s earliest thinking on the phenomena associated with the dilemma posed by a maltreating caregiver ([70], p. 642): “it is at least conceivable that some biologically-based strategy has been developed to deal with maltreating mothers … some parent-child conflict is to be expected, given the fact that parent and offspring are not genetically identical.” Given the prevalence of adversity in human experience, it seems inconceivable that humans would not have evolved a more active form of adaptation than disorganized attachment alone. Main’s original thoughts on internal working models are also worth considering in the context of these ideas ([71], p. 77): “While internal working models show a strong propensity for stability, they are not conceived as templates. They are best conceived as structured processes serving to obtain or to limit access to information.”

We find Main’s use of the word “information” here highly interesting, as we have suggested that limiting access to information may indeed be a biologically based strategy to deal with parental maltreatment, as described by Main. We have previously suggested that evolution may have “hijacked” the attachment relationship to use it as a channel for communications in relation to epistemic trust [72,73]. We have argued that epistemic mistrust—that is, closure towards the communication of social knowledge—may constitute an intergenerational adaptation to a harsh environment [74,75].

Humans are a social species, and to support our survival, natural selection has provided us with the tools necessary to interact and manage the complex terrain of our social world. We constantly and seemingly effortlessly infer the thoughts, feelings, and beliefs of other people, and much of our mental life is spent preparing to engage or engaging with the task of processing information about social agents [76]. A sociobiological picture that many researchers in the field have adopted is that the evolutionary advantages of living in relatively large social groups helped humans to evolve the capacities to interpret, explain, and predict each other’s actions, and to use these capacities to share and accumulate experiences, to plan, collaborate, and attain joint goals that they would not have been able to achieve as individuals or as members of smaller groups [77,78,79]. There are excellent neuroscientific experimental studies providing ample evidence that mentalizing, as indicated by the activation (or inhibition) of the brain’s mentalizing network (see “The neuroscience of mentalizing, attachment and resilience” below) is critical for assessing the morality of others, making judgements about how competitors might act and for learning from individuals with specialist information [80]. All these capacities would likely yield considerable selective advantage.

Early adversity, in particular complex trauma (i.e., negative experiences in early life involving neglect and/or abuse, typically in an attachment/caregiving relationship), has the potential to lead to severe impairments in mentalizing (for reviews, see [81,82]). The mentalizing model originated in understanding the psychological sequelae of trauma, with the assumption that a consequence of childhood adversity was a limitation of the capacity for mentalizing driven by anxiety. A fear of understanding the mental states of an individual who poses genuine threat to a child is understandable, and its generalization to other minds is to be expected [83]. Considerable evidence has accumulated to suggest that limitations in mentalizing are commonly associated with the experience of trauma, particularly post-traumatic stress disorder. Differences in mentalizing in traumatized individuals have been demonstrated on tests of emotional intelligence [84], tests of empathy [85], tests of compassion [86], and on tests specifically developed to measure cognitive mentalizing such as the Faux Pas test and the Strange Stories task [87,88]. It should be noted that our theoretical approach predicts a bias against internal cues to mental states (mental-state understanding) but a potential hypersensitivity to external indicators of mental states (observations) to balance the defensive scotomization of thoughts about what might be going on inside other people’s minds. In line with this prediction, traumatized patients appear to show no [89] or smaller [90,91] deficits in emotion recognition tests when compared with non-traumatized controls.

In the past, the authors have described impaired mentalizing as an outcome of the damaging effect of trauma on cognitive functioning [63,92]. Impaired mentalizing can also be seen as compromising the potential for social learning by making epistemic trust less achievable, and in this context, maintaining epistemic hypervigilance may even be an adaptation to an inherently untrustworthy social environment. In the face of maltreatment, it may make apparent sense to regard what other people try to convey as meaningless, unreliable or deceptive. However, as many authors writing about the impact of trauma point out, a strategy that might have survival value in the short term may turn out to cause significant difficulties later [93]. The problem with defensive ineffective hypermentalizing or hypomentalizing is that in the long term, the child may be unable to benefit fully from social learning: disrupted mentalizing may impede the emergence of the “epistemic match” between minds that is necessary to facilitate such learning [72]. The negative cascades described by attachment theorists [94,95] that flow from relationships in infancy and early childhood can be understood in terms of the level of connection an individual child feels they have in relation to their experience of the broader social environment.

But what of the intergenerational transmission of adversity? There is modest meta-analytic support for the intergenerational continuity of maltreatment (see [96,97,98]). However, the observed associations are often weak, have at most a medium effect size, and vary considerably across studies [97,99,100]. Some of the differences between findings may be accounted for by methodological discrepancies (e.g., the use of official records of offspring maltreatment versus self-reported abuse), but, on the basis of the theory advanced in this paper, we suspect that the transgenerational continuity of maltreatment rests in the context of social learning experienced by the parent and child. We would anticipate the transgenerational effects to be particularly large when the impact of maltreatment on parental mentalizing substantially interferes with the establishment of joint intentionality with the child.

The social learning deficit model would predict high levels of intergenerational transmission of trauma because if teaching and learning are social processes that depend on mentalizing, then individuals who are not open to social learning and find it difficult to mentalize will be less able to respond sensitively and to “teach” their children about the self and other. The developmental, interpersonal view on the emergence of epistemic trust in early relationships suggests a new perspective on attachment. The authors suggest that one of the advantages afforded by secure attachment is that it supports the capacity for social learning because it expedites epistemic trust [73], and this may account for some of the mental health benefits associated with secure attachment [12,13,14,101]. There is evidence from research to support the developmental nature of epistemic trust, and the social factors that influence the development and epistemic trust are consistent with an attachment theory approach [102,103,104,105]. Attachment relationships constitute a powerful source of information about the social environment and the extent to which it might be appropriate to pay attention to other people’s mental states [106]. The sense of “feeling wanted” does not rely on only one or two caregivers recognizing the intentionality of the child. We suggest that an evolutionary psychology perspective locates this experience far closer to the social network to which the child belongs.

The benefit of a secure attachment relationship, considered in relation to social groups rather than individuals [107,108,109,110,111,112], is that it enables the child to orient themselves toward opportunities for cultural learning provided by their environment [74,75]. This idea is encapsulated in Tomasello’s [113] proposal of collective intentionality; this is a form of shared intentionality in which the child coordinates their actions with an increasingly large social group that ultimately becomes their culture. This enables the child to communicate with, learn from and collaborate with those who are around them because the child shares with them a sense of identity obtained through common practices, beliefs, attitudes and a sense of belonging or identity.

This is not intended to downplay the importance of dyadic attachment. Sensitive communication by the caregiver [114,115,116] is likely to teach the child to attend to ostensive cues that trigger the “we-mode” [117]. The we-mode is a form of relational mentalizing that involves “recognition of the other persons as agents or persons just as real as oneself … the recognition of an inescapable fact that characterizes the human condition” ([113], p. 56). The we-mode may partly explain the educational advantages associated with secure attachment [118,119,120]. The authors suggest that sensitive caregiving engenders basic trust in the infant as the infant develops the expectation that the caregiver will respond with reasonable attunement to their needs and create experiences of shared intentionality. The caregiver’s demonstration of their accurate perception of the infant’s mental state creates a co-representation of the infant’s sense of self, shared by the caregiver and the infant, that supports epistemic trust. This ensuing epistemic openness supports exploration, the pursuit of joint intentionality, and learning from others: the we-mode is established as a source of consolation, support or interest. Thus, caregiver sensitivity fosters the capacity for social learning and adaptation across cultures. A series of studies from 2020 demonstrated the cross-cultural relevance of caregiver sensitivity by showing that it could be reliably identified in interactions in different cultures in Yemen [121], Iran [122], South Africa [123], Peru [124], Kenya [125], Indonesia [126] and Brazil [127].

In focusing on social learning, we consider that sensitive (or vulnerable) developmental periods extend into later childhood and adolescence. During these periods, children and adolescents have their perceptions of the world as safe and reliable, or the opposite, heightened in their experiences at school and in their widening social world. While early attachment to caregivers increases the chances of a positive outcome [128], processes other than early attachment may be activated in the social environment of the school and with peers. Support and acceptance from peers, rather than parents, is the best predictor of resilience in adolescence [129]. Nonetheless, the mechanisms we have identified in studying early development may be very helpful in understanding social influences later in life. Shared intentionality will be (or will not be) established with a range of social contacts, and the balance of the I-mode and we-mode will determine the individual’s openness to learning and influence in these contexts. Furthermore, epistemic trust can be understood as a generalized trust in one’s community. Shared intentionality may be experienced at a group level as well as at a dyadic level. In the group context, it involves the expectation that the social environment will protect and care for the individual and help them to achieve their goals and ambitions. It reflects the extent to which the cultural content of one’s community and the task of learning, teaching and contributing to that content is regarded as a shared and meaningful project.

## 4. The Neuroscience of Mentalizing, Attachment and Resilience

The psychosocial processes involved in achieving the we-mode, joint attention and epistemic trust are dependent on mentalizing processes. Mentalizing is necessary to help individuals process the social signals that are integral to successful social interactions, and it is assumed by many researchers to be a key factor in the etiology of significant psychosocial impairments commonly linked with mental disorders [130]. However, there is no single overreaching mentalizing mechanism. A network of brain regions supports a number of mental processes for representation, reasoning and control, and these processes link the mind of one person to the mind of another. The link may consist of joint attention established by following a person’s gaze, coordinating one’s actions with them through mimicry or imitation, taking their perspective in a social situation, trying to infer their state of mind, empathizing with their emotional experience or making judgements about their trustworthiness. The individual uses what they know about the other to generate an explanation of their actions (“he loves the theatre and Shakespeare especially, so he must dislike this director, which is why he did not come to the opening night of the play”). We have extraordinary capacities to simulate action even for situations that we have never experienced in order to judge whether someone’s reaction is “reasonable” or is beyond reason. We commonly go beyond stereotypical assumptions and simple extensions of our state to others and generate helpful pictures of subjective reactions that enable us to navigate challenging social situations. Yet, our capacity for mentalizing is also limited, which may be in part due to the multifaceted nature of adaptive (or full) mentalizing.

Neuroscientific research has indeed shown that mentalizing is not a unitary construct and relies on different supporting neural systems. Here, we briefly summarize the decades of research on the network of brain regions that has been shown to have the primary function of underpinning the human capacity for “mind-reading”. Understanding the brain mechanisms underpinning mentalizing illuminates the forms of ineffective mentalizing encountered by therapists in clinical work, and productive approaches to addressing these mentalizing inadequacies in therapy are clarified by an appreciation of the functioning of brain structures that support mentalizing. There is a large body of evidence supporting the existence of two distinct types of brain network underpinning social cognition: the mentalizing system (or Theory of Mind system) and the mirror neuron system (MNS). Whereas the MNS is probably engaged early in social information processing, the mentalizing system is activated when a person infers another’s intentions, irrespective of the specific task or stimulus.

Thousands of studies using modalities such as functional magnetic resonance imaging, event-related potentials, convergent transcranial magnetic stimulation, and transcranial direct current stimulation point to a role in mentalizing for a number of nodes in the brain, including the right and the left temporoparietal junction (TPJ), the posterior superior temporal sulcus (pSTS), the dorsomedial prefrontal cortex (dmPFC) and the precuneus [131]; there is also some evidence that the ventromedial prefrontal cortex (vmPFC) is involved [132]. Mentalizing appears to draw on a range of processes that are frequently activated in relation to specific mentalizing nodes [133]. For instance, the TPJ seems to have a role in surmising immediate states of mind (e.g., what someone currently believes, feels or wants). The STS has been associated with the recognition of biological movement and is also thought to be involved in the ability to adopt another person’s visual viewpoint and sensitivity to eye movements [134]: when movements in the Frith–Happé task of animated triangles elicited the perception of intentionality, there was an increase in connectivity between the V5, a brain region concerned with the detection of low-level motion, and the pSTS, which is part of the TPJ and is associated with biological motion [135]. This may be an example of feed-forward connections from sensory regions into the mentalizing system. In another study using animated shapes to generate the experience of intentionality, Moessnang et al. [136] reported that the pSTS was involved in the processing of social information based on perception, suggesting that the TPJ/pSTS responds to cues from sensory regions responsible for basic processing [137]. Further detailed work has revealed that these regions are likely to support mentalizing in part by encoding social prediction error; that is, they respond in particular when expectations are confounded [138], for example, when people appear from behind a barrier unexpectedly [139]. The right TPJ may have a special role in judgments of intentionality and culpability, affecting moral judgments [140] and also in activating mentalizing to explain why an agent might be deviating from behavior that would be predicted from their usual disposition [80]. Frith and Frith [137] suggest that the TPJ/pSTS receives information (“bottom-up” signals) concerning people’s behavior and is influenced by expectations (“top-down” signals) about the sort of behavior that would be expected.

The medial prefrontal cortex (mPFC), in turn, is the likely source of top-down signals, which are typical of voluntary, controlled actions. In an early study [141], mPFC activity marked conditions where participants believed that they were interacting with another person rather than a computer, even though there was no difference in the behavior of their partner; activity in the mPFC corresponds to tonic (mentalizing) activity. Multivariate and data-driven methods have suggested that areas in the mPFC have roles in processing familiar faces [142], in holding a model of the other’s mind [143], in tasks that involve reasoning about others’ false beliefs [144], in preferentially responding to spontaneous social interaction [145] and in encoding trait information [146].

The mPFC is also consistently linked to self-representation. The vmPFC is active, for example, when making trait judgments about the self in comparison with a familiar but not personally known person [147,148]. Thinking about the self generates more activity in the mPFC than thinking about a close friend [149], and the mPFC is active when using personal information to help decide about the preferences of another individual [150]. There is thus clear evidence that areas of the mPFC are involved in mentalizing both the self and others [151].

The prefrontal cortex is assumed to be involved in metacognitive representation [136]. It is possible that the same mPFC mechanisms are applied to the self and the other through the operation of metacognition, which is often defined as the “monitoring and control of cognitive processes”, potentially in relation to both the other and the self [137]. This has been demonstrated for decision making, where the processes underlying metacognition were shown to provide comparable models for monitoring one’s own decisions and for monitoring the decisions of others [152].

Other work has shown that sub-regions of the mPFC, including the vmPFC, the anterior cingulate cortex (ACC) and the adjacent paracingulate cortex (PCC), are also recruited for mentalizing [80]. The precuneus/PCC has also been associated with spatial navigation, scene processing, mental imagery, self-referential processing, multimodal first-person and third-person perspective-taking, the formation of impressions and episodic memory retrieval [131,153]. The involvement of the precuneus/PCC in spatial navigation indicates its function in mentalizing, as it has access to the same spatial information represented in different ways (e.g., a self-focused or an other-focused representation of space) so it can support switching between different perspectives on a scene [154] with its role in third-person perspective-taking [155]. From the authors’ perspective, a particularly interesting suggestion is that the same neural mechanisms may be used for navigating the social and physical spheres [156]. Social distance or social proximity may reflect the degree of difference in perspective and may explain the involvement of the PCC in the perception of social hierarchy [157].

In a large activation likelihood estimation meta-analysis of 144 datasets (comprising 3150 participants) [158], the mPFC and bilateral TPJ were identified as being recruited during Theory of Mind tasks. Prefrontal areas are clearly involved in making inferences about the enduring dispositions of a social target, whereas self-referential brain activity appears to be localized to the vmPFC [159] and also the dmPFC [160]. The temporal poles appear to be involved in semantic memory of social contexts and scripts, the retrieval of autobiographical episodic memory, and personal semantic memory, including socially relevant processes such as the recognition of familiar faces and voices, the processing of emotions, and linking emotional responses to stimuli, including third-person perspective-taking in emotional contexts [134,161]. Experiences of social rejection appear to demand activity from the mentalizing network [162,163].

The default mode network (DMN) is active when the mind is not engaged in any specific cognitive task (i.e., when the person’s mind is “wandering”). There is substantial evidence that this is reciprocally suppressed by the task-positive mode when individuals perform a purposeful activity. The DMN overlaps with and extends beyond the mentalizing network. Jack et al. [164] have shown that the DMN is activated by a set of tasks concerned with social cognition (i.e., reasoning about the mental states of others).

Specific neural activity related to the observation of action is seen in a network of cells designated mirror neurons, which were first observed in monkeys [165]. The MNS fires either when a monkey performs an action or when the monkey watches another monkey performing the same action. This indicates that the brain can bring into alignment what ones sees with what ones does [166]. The human MNS consists of the ventral premotor cortex, inferior parietal lobule, and superior temporal sulcus. These regions are activated when actions—including facial expressions—are both performed and observed [167]. Some of these regions are also involved in empathy [168]. The location of these neurons in the motor system, rather than the perceptual system, suggests that the brain makes inferences about the goals of the actions of others that it observes “imagines itself” performing those actions [169]. The MNS enables an individual to make inferences about why someone is doing something based on why the individual might, in some circumstances, perform the same action. The role of the MNS in mentalizing has been clarified by several studies exploring the rapid spatiotemporal dynamics of mentalizing using neuroimaging modalities with high temporal resolution but coarser spatial resolution (source-space electroencephalography and magnetoencephalography) [170]. The consistent observation is that the spatiotemporal sequence commences in the visual cortex, followed by the MNS regions, and with the DMN regions last in the sequence [171,172]. This suggests that the MNS identifies observable actions, which are then used by the mentalizing network to infer unobservable mental states (e.g., observing the quality of a grasping action by an individual and making a judgment, based on the action, about the individual’s state of hunger) [173,174].

Thus, the MNS and the mentalizing system appear to have complementary [175] and sometimes also antagonistic roles. Specifically, automatic imitation (mimicry) is suppressed by the activation of the mentalizing network [176]. The MNS seems to set up an automatic identification with the social partner, which needs to be actively suppressed if an individual is to be able to separate sufficiently from the partner’s perspective to identify deception by the partner [177]. For example, the neutral, almost immobile, stance of the classical psychoanalytic therapist may make more sense if the MNS is regarded as perhaps priming an unboundaried closeness to an individual’s conversational partner’s perspective that may deny them the benefit of the individual’s objectivity and independent insight. By suppressing mimicry and imitation, the therapist sharpens their sensitivity to the unique thoughts and feelings of their client, minimally contaminated by their own experience.

There may be further important aspects of the hierarchical organization of the mentalizing system that ensure the possibility of affecting processing by top-down activation or inhibition. For example, viewing pictures that are unpleasant will elicit amygdala activity regardless of the task the viewer is asked to undertake (e.g., to rate the subjects of the images for their physical appearance or scariness), but a task that demands introspection (e.g., a task requiring the participant to rate the emotion evoked by a stimulus) will elicit mPFC and precuneus/PCC activity regardless of the picture content, directing attention to internal states [178]. By contrast, activity in the anterior insula was found to be increased when an unpleasant stimulus was presented to participants in a task that demanded introspection [179]. This is consistent with the assumption that the anterior insula integrates bottom-up interoceptive signals with top-down signals to create a state of emotional awareness, with top-down signals from the mPFC during introspection enhancing the anterior insula response to bottom-up signals of unpleasantness from the amygdala [137].

As we look at mentalizing in greater detail the temptation is to identify specific processes and sub-processes, and in so doing to lose sight of human social understanding as an indivisible general process in terms of its output. Mentalizing activities, however they might be subdivided, entail core brain processes which involve making use of previously acquired knowledge that is retrieved and manipulated essentially as concepts [180]. It has been suggested that in some ways, mentalizing can be thought about not as entailing particular activities, such as remembering the past, envisioning the future and making plans for it, reflecting on the self, making moral judgments, interpreting and predicting the behavior of others and “daydreaming”, but rather as using highly evolved generic brain processes for the purposes of acquiring, storing, retrieving, selecting, analyzing and synthesizing representations of concepts, which are defining features of the human brain [180]. Mentalizing may entail specific brain processes, but its integrated achievements cannot be separated from perceiving, thinking, acting, or just “being a person”. These processes, in combination, enable humans to reflect on themselves and others, and to reach out to others, particularly when faced with adversity. Most importantly, beyond attachment, they also enable the broader salutogenic processes associated with solid mentalizing, and thus ultimately facilitate the development of what we might call resilience, associated with epistemic trust.

## 5. Conclusions

In our developmental approach to trauma, we have previously suggested that adversity is traumatic when it is accompanied by a sense that the mind is alone. Normally, the presence of another’s mind provides social referencing that helps an individual to frame a frightening experience that would otherwise be overwhelming [83]. Recent experimental studies have provided strong support for this view. Studies of conditioning have revealed that vicarious safety learning (when participants observe a calm demonstrator modelling safety) leads to better attenuation of a conditioned threat response than with traditional direct safety learning in which a model is not present [181]. This suggests that the human mind is programmed to be attuned to socially accessible agents to judge the response to threat through the availability of another person.

An initial adaptation to a harsh environment is to seek out help (i.e., involving the activation of the attachment behavioral system) in the first instance, but if appropriate help is not received, epistemic vigilance may be a further adaptation. The issue stops being one of attachment-seeking behaviors and becomes something more diffuse: an adaptation to social circumstances that involves heightened awareness of the need to assess the social environment and its cues. Emerging empirical research indicates that disruption in the epistemic stance is indeed associated with exposure to early adversity [182]. A stronger insensitivity to ostensive cues is an adaptation to a harsh environment [72]. The relationship between maltreatment and psychopathology is real [183]. Epistemic vigilance carries risks with it, but it may be the most effective strategy in particular environments. A particularly stark example of this is the elevated prevalence of schizophrenia in migrant communities, which suggests that schizophrenia might be understood, in part, as a maladaptive outcome of certain adaptations to being in a hostile environment [184]. The Bayesian idea is important here: a sense of cumulative learning and adaptation, which in some people, in conjunction with other vulnerabilities, may tip over into severe disorder.

In moments of adversity, we all experience the pain of feeling unaccompanied by another mind. However, if we are functioning reasonably well and live in a sufficiently benign environment, experiences of adversity increase motivation to identify sources of social connection and support. These connections, in turn, ideally help to restore mentalizing, fostered by and further fostering epistemic trust in a “we-mode”, which mitigates the traumatic isolation associated with adversity. If such a process is not possible, a more pernicious cycle unfolds: the experience of adversity generates and/or activates epistemic mistrust, which creates isolation and the experience of an absence of social support. Epistemic hypervigilance is then triggered by the subjective experience of shame, social exclusion, and social alienation. The epistemic system is exhausted, leading to a non-discerning state in which the individual may veer between epistemic isolation and indiscriminate trust—either of which will increase social dysfunction and vulnerability.

To survive in our social and connected world, we have to be able to learn from others [185]. In learning the skills we need to survive, we observe and listen to experts; we avoid punishments and receive rewards by imitating others; we make complex strategic decisions having taken others’ experience and views into account; and all along we learn about ourselves through social interactions. Learning efficiently from others is a central part of human existence. As Espinosa et al. [186] point out, the transmission of information from parents to children, and between peers, is a core mechanism of adaptive cultural learning. However, it can also result in maladaptive behaviors such as antisocial actions, exaggerated avoidance, and anxiety [186]. As we have described in this review, a series of highly specialized and multilayered neural regions is involved in these processes and underpins the capacity for mentalizing and for epistemic trust, and thus the development of resilience. The importance of social networks in mental health may thus have been hiding in plain sight. Attachment as a construct has traditionally always been linked to close relationships, whether between a child and their caregivers or between partners in a romantic relationship. In our view, it should be extended to relationships within the social environment.
